# Assessing Nicotine Exposure in Users and Non-Users of Electronic Cigarettes Through Silicone Wristbands

**DOI:** 10.3390/ijerph22030388

**Published:** 2025-03-07

**Authors:** Giovanni E. Appolon, Samantha Suess, Alice Xayavong, Nicolas Lopez Galvez, Nathan G. Dodder, Eunha Hoh, Penelope J. E. Quintana, Eyal Oren

**Affiliations:** 1 School of Public Health, San Diego State University, San Diego, CA 92182, USA; ssuess@sdsu.edu (S.S.); axayavong4401@sdsu.edu (A.X.); nilopez@sdsu.edu (N.L.G.); ehoh@sdsu.edu (E.H.); jquintan@sdsu.edu (P.J.E.Q.); eoren@sdsu.edu (E.O.); 2 Herbert Wertheim School of Public Health and Human Longevity Science, University of California San Diego, La Jolla, CA 92093, USA; 3 Research Foundation, San Diego State University, San Diego, CA 92182, USA; ndodder@sdsu.edu

**Keywords:** exposure assessment, particulate matter, silicone samplers

## Abstract

Although the use of electronic nicotine delivery systems (ENDS) among youth has declined since its peak in 2018, it remains popular among young adults. Despite its popularity, research on the health effects of secondhand exposure to ENDS remains limited. Silicone wristbands offer a simple, cost-effective method for measuring nicotine exposure. The study employed a quasi-experimental design and recruited six dyads consisting of ENDS users and non-users. Over three consecutive months, all participants wore silicone wristbands for one week at a time to assess nicotine exposure. ENDS users had a higher overall median nicotine concentration in their silicone wristbands (423.2 ng/g, IQR: 199.2–669.1) compared to non-users (17.2 ng/g, IQR: 6.5–128.0). This trend was consistent across all time points. Statistically significant differences between ENDS users and non-users were observed during months 1 and 2 (*p*-values = 0.0303 and 0.0411, respectively), but not during month 3 (*p*-value = 0.2468). Similar trends were observed in urinary cotinine levels, with higher medians among ENDS users (1013.0 ng/mL, IQL: 442.0–1490.0) compared to non-users (1.3 ng/mL, IQL: 1.0–1.4). A significant correlation was found between urinary cotinine and wristband nicotine levels only in non-users (r = 0.69, *p*-value = 0.0017). Silicone wristbands worn by non-users can detect secondhand nicotine exposure and are significantly correlated with urinary cotinine.

## 1. Introduction

In the past decade, the risks and benefits of electronic nicotine delivery systems (ENDS) have been a subject of debate among tobacco researchers [1]. Despite ENDS recognition as a form of nicotine replacement therapy that can assist in smoking cessation [1,2], significant concerns have emerged in the United States due to the growing trend of teenagers and young adults, with no history of cigarette use, adopting ENDS products [3]. Although ENDS use among youth in the United States peaked in 2018 at 27.5% and has since declined to 7.8% in 2024, the frequency of use among current ENDS users has increased [4]. As of 2024, 38.4% of current ENDS users in the U.S. reported using ENDS at least 20 times in the last 30 days, and 26.3% reported using ENDS products daily [4]. In Great Britain, the proportion of ENDS users aged 11–17 increased from 4.0% in 2021 to 14.1% in 2022, according to recent research [5]. Additionally, data suggest that ENDS use among adolescents is more prevalent in the U.S. and Europe compared to Asian countries and Brazil [5].

Although ENDS are considered less harmful than traditional cigarettes, they are not without risk to human health. Some examples of potential health risks associated with ENDS use among youth include poor brain development and development of severe lung injury, such as bronchiolitis obliterans [6,7]. In 2019, an outbreak of 805 cases of lung injury associated with ENDS emerged, which led to 96% of the cases being hospitalized, and the first description of electronic cigarette or vaping use-associated lung injury (EVALI) [8,9]. Later, it was understood that vitamin E acetate, a synthetic form of vitamin E found in some ENDS products, was strongly associated with EVALI [10].

In addition to the risks ENDS users face, secondhand exposure to high concentrations of particulate matter (PM_2.5_ particles with aerodynamic diameters ≤ 2.5 µm) and nicotine emitted by ENDS’ aerosol can also increase the risk of poor health outcomes among non-ENDS users [11]. Prior studies have indicated that ENDS use can raise PM_2.5_ levels to as high as 1121 µg/m^3^, nearly 45 times higher than the World Health Organization’s recommended limit of 25 µg/m^3^ for 24 h exposure [11,12]. Exposure to high levels of PM_2.5_ is associated with an elevated risk of developing chronic cardiovascular and respiratory diseases [13]. Additionally, the average concentration of nicotine emitted by ENDS users is about 3.32 µg/m^3^ [14]. Secondhand exposure to ENDS has been associated with an increased risk of developing bronchitis symptoms and shortness of breath among young adults [15]. Given the potential health implications associated with exposure to ENDS, it is important to assess vulnerable populations at risk and determine where they are being exposed.

The three main approaches that are normally used to evaluate environmental tobacco exposure consist of measuring tobacco smoke components in the air, obtaining self-reported use of tobacco through questionnaires/interviews, or obtaining tobacco-specific biomarkers through urine or blood samples [16]. Each of these approaches has its limitations. Air sampling for compounds derived from tobacco smoke is reliable; however, active air sampling for personal exposure assessment, which has been the most frequently used method in prior studies, may be too restrictive for study participants, especially for children, as it requires an air pump and collector [17]. Questionnaires and interviews are subject to recall bias. Finally, measuring tobacco-related biomarkers from biological samples such as blood or urine samples may be difficult, as collecting these samples from a child or young adult is costly and inconvenient due to embarrassment or perceived lack of benefit [17]. To overcome these limitations, studies often use a combination of these methods to provide a more comprehensive assessment of tobacco exposure. However, utilizing multiple methods can be inconvenient for researchers, as it often requires additional time, resources, and coordination between different data collection strategies.

A possible solution to address these limitations is to use silicone wristbands. Silicone wristbands are a low-cost tool with the potential to be utilized as a passive sampler to detect a variety of toxic chemicals, such as pesticides and phthalates [17,18]. Although silicone wristbands also can detect nicotine exposure [17], only two studies have utilized silicone wristbands as a passive sampler to measure nicotine levels, and both studies evaluated secondhand exposure among children [17,19,20].

The objective of this study was to understand the potential for secondhand ENDS exposure among non-users and to utilize and compare biological samples with silicone-based wristband samplers to evaluate secondhand exposure to ENDS among college students.

## 2. Materials and Methods

A total of six dyads (12 participants) were recruited for the study. Among these dyads, three were roommates, while the remaining three were close friends who spent time together almost every day. Each participant was provided with silicone wristbands and followed up over three months. During the second week of each monthly visit, silicone wristbands, urine samples, and survey questionnaires were collected from each participant.

### 2.1. Recruitment

The study was conducted at San Diego State University (SDSU). Institutional Review Board (IRB)-approved flyers (IRB Protocol Number HS-2020-0071) were distributed on the SDSU campus and uploaded to social media platforms (e.g., Twitter and Facebook), as well as the ResearchMatch website. ResearchMatch is a national health volunteer registry created by several academic institutions and supported by the U.S. National Institutes of Health as part of the Clinical Translational Science Award program [21]. It has a large population of volunteers who have consented to being contacted by researchers about health studies for which they may be eligible [21]. The flyers contained a brief description of the study and a quick response (QR) code that directed potential ENDS user participants to an eligibility survey. The eligibility survey and QR codes were created using Qualtrics XM [22].

Dyads were recruited for this study, consisting of ENDS users and non-users. Two eligibility questionnaires were created to obtain potential ENDS user and non-user participants. The ENDS user questionnaire aimed to recruit individuals who currently use any form of vaping products, as advertised on IRB-approved flyers. Eligible ENDS users had to provide informed consent, be aged 18 to 30, actively use e-cigarettes for a minimum of 25 days each month, consume at least three e-cigarette pods a week, or an equivalent of two milliliters a week, use e-cigarettes for nicotine at least 50% of the time, fluent in English, and willing to wear a silicone band for one week at a time. Exclusions for ENDS users included recent use of alternative tobacco products, participation in a smoking cessation program, use of nicotine replacement therapy/medication for vaping cessation, or attempts to quit/cut down on e-cigarette use in the past month.

Eligible ENDS users were asked to provide contact information for a roommate or close friend as their non-user counterpart in the dyad study. Non-users of ENDS were considered eligible if they did not plan to become users and had daily contact/interaction with the participant who qualified as an ENDS user. Non-user participants had to be aged 18–30 years old, agree to wear a silicone wristband, and speak fluent English. Once both ENDS users and non-users were deemed eligible, they were invited to attend an in-person interview.

### 2.2. Sample Collection

All eligible dyads were contacted by research assistants by email or text message. The study involved six sessions conducted over three months. In the initial session of each month, participants were provided with silicone wristbands to wear consistently for a minimum of seven days. Participants were followed up a week later to return the wristbands, answer survey questions, and provide urine samples. Subsequently, participants were scheduled to return 30 days later, and the process was repeated for months two and three. The first session of each month lasted approximately 15 min, while the second session of each month lasted approximately 30 min. At the end of each month, all participants received Amazon gift cards as compensation in $25, $50, and $75 increments.

### 2.3. Interview Process

All participants were asked for demographic information, current health status, perceptions of electronic cigarette use, and secondhand exposure to vapor from ENDS using a survey that was created in Qualtrics. ENDS users were asked additional questions regarding their vaping habits. The same questionnaire was administered to participants in months 2 and 3, with the exclusion of demographic information already provided in month 1.

### 2.4. Nicotine and Cotinine Measurement Methods

The sample preparation and isotope-dilution liquid chromatography tandem mass spectrometry (LC-MS/MS) methods for the quantification of nicotine in the silicone wristbands and cotinine in urine have previously been described in detail [17]. Briefly, wristbands were cut into pieces, were spiked with the internal standard nicotine-*d*_4_, extracted using QuEChERS [17], and syringe filtered. Nicotine was quantified by LC-MS/MS using hydrophilic interaction liquid chromatography (HILIC) and positive electrospray ionization (ESI+). One quantitative and two confirmation multiple-reaction-monitoring (MRM) transitions were used for nicotine detection. The limit of quantification (LOQ) and estimated method detection limit (MDL) for nicotine was 0.30 ng of nicotine/wristband and 0.19 ng of nicotine/wristband, respectively. Wristband weights vary, but average approximately 4 g, so for a 4 g wristband, the MDL would be 0.07 ng nicotine/g silicone and 0.05 ng/g silicone, respectively. Urine samples were spiked with the internal standard cotinine-*d*_3_, extracted by an alternate QuEChERS method, and syringe filtered. LC-MS/MS quantification of cotinine used reverse phase C_18_ chromatography and the ESI+ mode. Cotinine detection by MRM used one quantitative and two confirmation transitions. The LOQ and MDL for urinary cotinine were 0.10 ng/mL and 0.033 ng/mL, respectively.

### 2.5. Statistical Analysis

Descriptive statistics included geometric means with 95% confidence intervals, medians, interquartile ranges (IQR), and minimum and maximum values for both the silicone wristband nicotine and urinary cotinine data from each month. A non-parametric Wilcoxon test was conducted on the raw data to assess whether a statistically significant difference existed between ENDS users and non-users for both the wristband nicotine and urinary cotinine levels. The test was performed on the overall sample as well as on each individual time period. To achieve a normal distribution, a natural log transformation was applied to both nicotine wristband and urinary cotinine values. A “+1” constant was added only to the urinary cotinine values to account for zero values before the natural log transformation. A bar graph of the median natural log-transformed values for both the silicone wristband nicotine and urinary cotinine was created to show trends in median values over time. Dual-axis line graphs were created for both ENDS users and non-users to evaluate individual trends in nicotine measures from wristbands and urine across all time points. Pearson correlation tests were conducted to assess the relationship between the natural log value of urinary cotinine and the natural log value of nicotine in silicone wristbands, stratified by ENDS use.

Secondhand exposure to vapor from ENDS was assessed by utilizing the survey questions that asked participants, “In the past seven days/24 h, where were you exposed to secondhand vapor?” The response options included: at home, at a friend’s house, at a relative’s house, in a vehicle, in public areas, and somewhere else. Participants had the option to select all that applied. Clustered bar graphs were constructed to visualize the overall distribution of exposure locations and the distribution of exposure locations by vaping status. The total number of “yes” responses across all exposure locations provided by the 12 participants throughout the three-month study period for both exposure questions (exposure within the past seven days and exposure within the past 24 h) was calculated. To determine the most frequent locations where participants were exposed, the number of “yes” responses for each exposure category was divided by the total number of exposures reported by the participants. Additionally, to determine the percentage of ENDS users and non-users who responded “yes” to being exposed in each category, the number of users and non-users for each category was divided by the total number of “yes” responses. RStudio version 4.4.2 (Posit PBC, Boston, MA, USA) was used for all analyses, and the ggplot2 (Hadley Wickham, New York, NY USA) package was utilized to create all figures.

## 3. Results

Table 1 presents descriptive statistics for nicotine exposure, measured through silicone wristbands and urinary cotinine levels. Among ENDS users, the overall geometric means were 324.1 ng/g (95% CI: 195.3–537.8) for nicotine in silicone wristbands and 759.3 ng/mL (95% CI: 474.0–1215.0) for urinary cotinine. For non-users, the geometric means were 27.9 ng/g (95% CI: 12.9–60.8) for nicotine in silicone wristbands and 1.7 ng/mL (95% CI: 1.1–2.7) for urinary cotinine. ENDS users consistently had higher geometric means of nicotine exposure compared to non-users. Specifically, ENDS users had nicotine wristband geometric means of 488.9 ng/g (95% CL: 281.4–849.4) in month 1, 415.7 ng/g (95% CL: 229.8–752.1) in month 2, and 159.3 ng/g (95% CL: 46.3–548.5) in month 3, indicating a decreasing trend in nicotine exposure over time. Similarly, non-users had nicotine wristband geometric means of 37.1 ng/g (95% CL: 8.7–158.2) in month 1, 37.8 ng/g (95% CL: 6.6–216) in month 2, and 15.6 ng/g (95% CL: 7.1–34.2) in month 3, also showing a decreasing trend over time. In contrast, the trends for urinary cotinine levels varied. For ENDS users, the geometric means of urinary cotinine were 846.0 ng/mL (95% CL: 327.9–2188.4) in month 1, 753.0 ng/mL (95% CL: 243.6–2315.0) in month 2, and 686 ng/mL (95% CL: 234.0–2017.0) in month 3. For non-users, the geometric means were 1.4 ng/mL (95% CL: 0.9–2.1) in month 1, 2.9 ng/mL (95% CL: 0.6–13.7) in month 2, and 1.2 ng/mL (95% CL: 1.0–1.4) in month 3. A non-parametric Wilcoxon test was conducted to assess statistical differences between ENDS users and non-users. A significant difference in nicotine wristband levels was observed overall and during months 1 and 2 (*p*-values = 0.0005, 0.0303, and 0.0411, respectively), but not in month 3 (*p*-value = 0.2468). In contrast, urinary cotinine levels were significantly different between groups overall and across each of the three months (*p*-values = <0.0001, 0.0050, 0.0050, and 0.0049, respectively).

**Figure 1:**
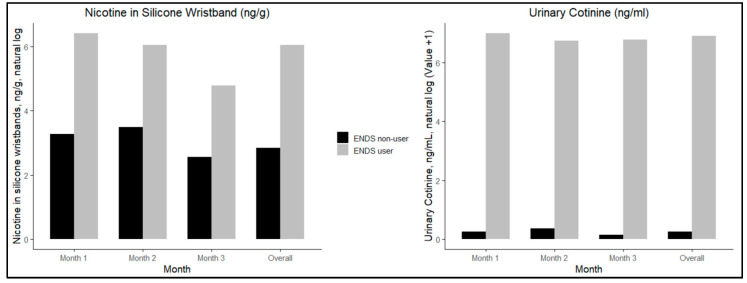
Median levels of natural log nicotine in silicone wristbands (ng/g) and natural log of urinary cotinine (ng/mL) across study months and overall.

Figure 1 presents log-transformed median trends for silicone wristband nicotine and urinary cotinine values. The figure demonstrates that the difference in nicotine in wristbands for exposed non-users as compared to ENDS users is lower than the difference in urinary cotinine, and that values slightly decrease in month 3.

**Figure 2:**
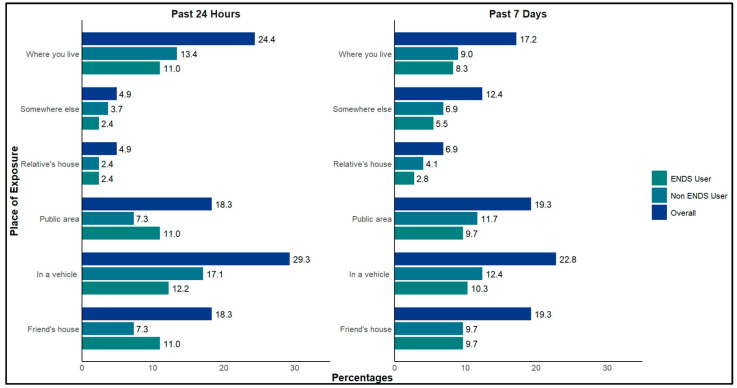
Percentages of self-reported physical areas of exposure. Exposure locations were tallied based on “yes” responses over the three-month period. The percentage of “yes” responses for each location was then calculated, and the proportion of users and non-users was analyzed. There were a total of 145 responses to exposure within the past seven days and 82 responses of exposure within the past 24 h across all time points.

Figure 2 provides the responses from participants who self-reported their exposure locations in the past 24 h and seven days during all follow-up visits throughout the three-month duration of this study. Each participant was given the option to select all applicable locations where they had been exposed. This resulted in a total of 145 responses of location-based exposure in the past seven days and 82 responses in the past 24 h within the three-month duration of the study. The most frequent location for exposure occurred in a vehicle (29.3 in the past 24 h and 22.8 in the past seven days) (Figure 2).

**Figure A1:**
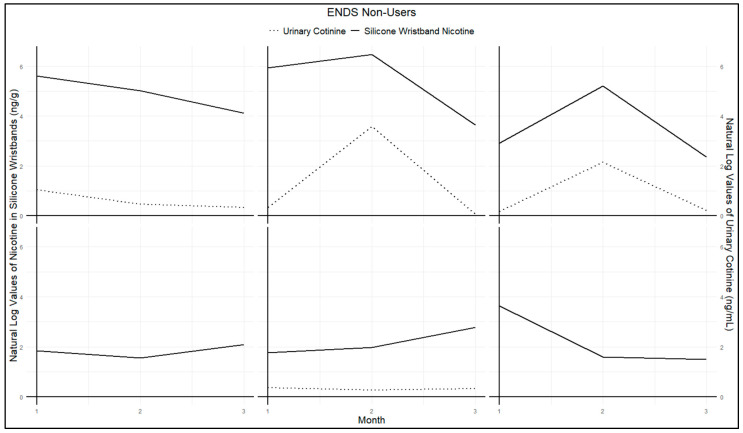
Individual silicone wristband nicotine and urinary cotinine trends for all e-cigarette non-users across all study time points.

**Figure A2:**
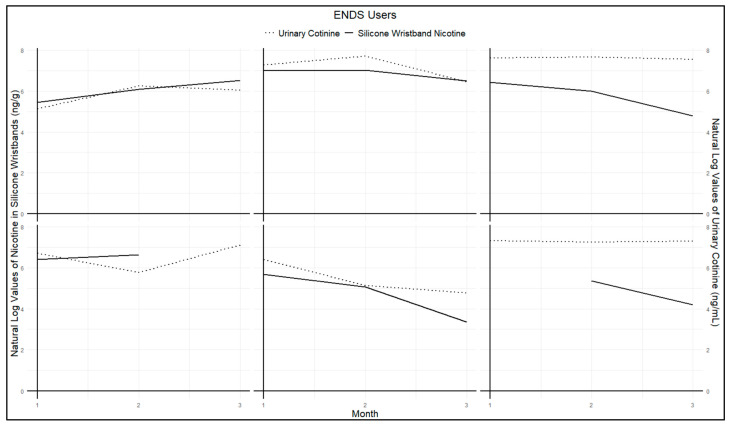
Individual silicone wristband nicotine and urinary cotinine trends for all e-cigarette users across all study time points.

**Figure A3:**
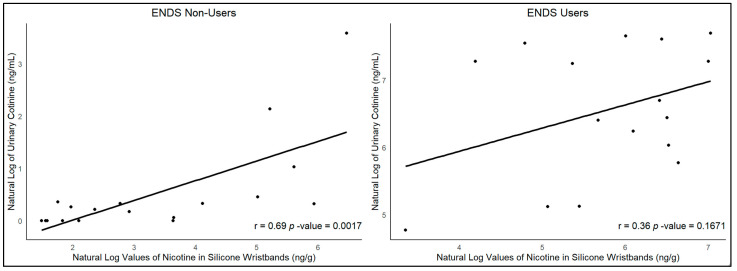
Pearson correlation between log-transformed urinary cotinine and log-transformed silicone wristband nicotine by vaping status.

The data presented in Table A1 and Figure A1, Figure A2 and Figure A3 can be found in the Appendix A. Table A1 illustrates the self-reported locations of secondhand exposure in each month from all individuals in the study. Results show that all non-ENDS users consistently reported exposure to ENDS vapor within the past seven days at each time point. Among ENDS users, secondhand exposure to ENDS in a vehicle was reported in 83% of all possible responses across all time points. A similar trend was observed for exposure in public areas, with 94% of all possible responses from non-ENDS users and 77% from ENDS users indicating secondhand exposure.

Figure A1 and Figure A2 present urinary cotinine measures and nicotine exposure via silicone wristbands for each participant in the study. Both figures demonstrate similar trends among non-users (Figure A1) and users (Figure A2). Specifically, high values of wristband nicotine exposure corresponded with high urinary cotinine concentrations, while low values of wristband nicotine exposure corresponded to low urinary cotinine levels. Parallel trends were observed for almost all participants. Some data points are missing in Figure A2 due to one participant losing their wristband at a single time point, and another wristband was excluded due to extremely high values. This wristband was returned several weeks later, outside the study’s required one-week return period, which likely resulted in unusually high values compared to other participants, leading to its classification as an outlier.

A positive Pearson correlation between urinary cotinine and silicone wristbands was observed (r = 0.69, *p*-value = 0.0007, Figure A3) among ENDS non-users. Among ENDS users, the results indicated no significant correlation between urinary cotinine and silicone wristbands (r = 0.36, *p*-value = 0.1671, Figure A3).

## 4. Discussion

Our results revealed higher geometric means and medians of nicotine exposure among ENDS users compared to non-users across all time points, as measured by silicone wristbands and urinary cotinine levels. Statistically significant differences between ENDS users and non-users were observed at most time points for both urinary cotinine and nicotine wristband measurements. Our results indicated a direct significant relationship between urinary cotinine and nicotine levels in silicone wristbands among non-vaping participants (r = 0.69, *p* = 0.0017). However, no such correlation was observed among ENDS users. The correlation among non-users might be due to the variability in ENDS exposure, reflected in both silicone wristbands and urinary cotinine levels. Such variability makes it easier to detect and test for a relationship. In contrast, ENDS users, who were all current or frequent users of ENDS according to our eligibility criteria, had less variability in nicotine exposure. This lack of variability made it challenging to assess the relationship between nicotine levels in silicone wristbands and urinary cotinine among ENDS users. However, nicotine levels in silicone wristbands were a median of 25-fold higher in ENDS users, whereas urinary cotinine levels were a median of 772-fold higher. This implies that nicotine in the wristband may arise mainly from exposure to airborne secondhand vape, whereas the cotinine in urine may arise from direct conversion from inhaled nicotine in the vape to cotinine in the body, excreted in the urine. The direct significant relationship between urinary cotinine and nicotine levels in silicone wristbands among non-vaping participants, not observed among ENDS users, suggests that silicone wristbands are effective in measuring secondhand exposure among non-vaping users.

Notably, among the various secondhand exposure locations assessed, exposure in a vehicle was reported most frequently, accounting for 22.8% of all “yes” responses for all exposure locations within the past seven days as shown in Figure 2. This trend was especially evident among non-users, who consistently reported vehicle exposure within the past seven days across all time points as shown in Table A1.

Self-reported data revealed that secondhand exposure to ENDS vapor was most commonly reported in vehicles, with 83% of possible responses from ENDS users and 100% from non-ENDS users. Public areas were also frequently cited, with 94% of possible responses from non-ENDS users and 77% from ENDS users reporting exposure. In contrast, exposure at a relative’s home was less common, accounting for only 33% of total responses from non-ENDS users and 22% from ENDS users across all time points.

Literature has shown that silicone wristbands mimic the way skin absorbs toxic chemicals, such as pesticides and phthalates, into the body [17,18,23]. Although silicone wristbands have the ability to absorb toxic chemicals, to our knowledge, only two studies have utilized silicone wristbands as a passive sampler to measure nicotine levels, and both studies evaluated secondhand exposure among children [17,19,20]. Results for both studies were similar to our findings, where a significant correlation was observed between urinary cotinine and wristband measurements [17,20]. Quintana et al. found that children exposed to secondhand aerosol from ENDS via caregivers had a median nicotine level of 27.6 ng/g, as measured from silicone wristbands [20]. This level was higher than the median of 17.2 ng/g observed in non-ENDS users in our study. This discrepancy may be attributed to differences in study populations. The study by Quintana et al. included participants aged 3 to 14 years old living at home with their caregivers, [20] whereas our study focused on college students aged 18 to 30. The higher nicotine measurement in the Quintana et al. study could be due to the closer and more frequent contact between children and their caregivers leading to increased exposure levels. In contrast, our participants, consisting of best friends and roommates, may spend less time together, resulting in lower exposure intervals. Our study also observed high reports of secondhand exposure in public areas. This finding aligns with a previous study from the National Youth Tobacco Survey, which reported that one in three middle and high school students have been exposed to secondhand tobacco smoke in public areas [24].

Our study contributes to existing literature by addressing the limited research on using silicone wristbands to detect secondhand nicotine exposure and by being the first study to employ a dyad approach for evaluating nicotine exposure among college students using this method. Given that prior studies have indicated that ENDS can increase indoor PM_2.5_ levels up to 1121 µg/m^3^ and nicotine levels in the air up to 3.32 µg/m^3^, employing a low-cost and feasible measurement tool capable of assessing these exposures is crucial for reducing poor health outcomes that are associated with secondhand exposure.

Several limitations should be acknowledged within our study. First, we had a small sample size of 12 participants (six dyads). The sample size makes it difficult to generalize estimates regarding the location of exposure among college students. Despite this limitation, we were able to observe a significant relationship between wristbands and urinary cotinine measures for non-users. An extension of this limitation is that we excluded one participant’s nicotine wristband result, as they returned their wristband several weeks late, resulting in unusually high values. This exclusion led to fewer participants contributing to the descriptive statistics for month 3, and we believe this reduction in sample size accounts for the observed changes.

Second, our study depended on eligible ENDS users to identify potential non-users for recruitment. Both ENDS users and non-users needed to meet our eligibility criteria to be included in the study. Consequently, if an eligible ENDS user could not find a matching non-user, they were unable to participate. This limitation prolonged the recruitment process into the summer session where there was less activity on the college campus for recruitment.

Third, half of our dyads were roommates, while the other half were close friends. We did not measure how frequently they spent time together, and given our already small sample size, variations in contact time between dyads may have influenced our results. Fourth, a minor issue arose during the study when one participant misplaced their wristband during the third-month visit, returning it a month later. Consequently, data from this wristband, which was in possession for an additional three weeks compared to others, was excluded from the study’s analysis as a major outlier in its value. Our final limitation was our inability to assess adherence to the continued wearing of the wristband. It is possible that the single participant did not wear the wristband consistently over the seven days, which could have led to inaccurate exposure measurements.

The final limitation was the use of urinary cotinine as a biomarker. Urinary cotinine has a half-life of 16–24 h and can detect nicotine exposure within two to four days post-cessation [25,26]. The wearing of wristbands for a minimum of seven days before collection has the potential to have an impact on external validity. Specifically, instances where significant exposure occurred over a weekend, yet wristbands were not retrieved until five days later, may have resulted in elevated nicotine levels detected by wristbands but lower levels by urinary cotinine.

Despite these limitations, a strength of this study is the use of a dyadic design to measure nicotine exposure among participants who spend the majority of their time together (i.e., best friends, roommates). Previous studies that utilized dyadic designs have provided evidence that harm resulting from secondhand exposure is more prevalent among non-tobacco users who maintain close relationships with tobacco users [27,28]. These studies have employed a mother–child/adolescent dyadic design, where mothers are tobacco users and children/adolescents are non-users [27,28]. While a mother–child dyadic design may be appropriate for assessing secondhand exposure among children, as they are more likely to experience in-home exposure [29], this approach may not be suitable for young adults, who are more likely to encounter secondhand exposure in public or other non-home environments. According to the National Youth Tobacco Survey, more than half of middle and high school students have self-reported secondhand exposure to tobacco products in both indoor and outdoor public places [30]. We overcame this gap in the literature by using best friend and roommate dyads for our analysis and obtaining self-reported information on exposure location within the past seven days and 24 h.

## 5. Conclusions

To our knowledge, this is the first study to utilize silicone wristbands to evaluate secondhand exposure to nicotine from ENDS among college students. Our study provides valuable evidence that silicone wristbands can serve as reliable and cost-effective tools for measuring secondhand exposure to ENDS among non-users. Future directions for this research include conducting analyses with larger sample sizes and incorporating information on the frequency of ENDS product use to examine its correlation with nicotine levels measured by silicone wristbands. Additionally, since more than half of middle and high school students have reported secondhand exposure in public areas [30], future studies should evaluate the locations where exposures to ENDS are most prevalent in order to inform policymakers about the areas that need attention to reduce secondhand exposure. Given our findings indicating secondhand exposure to ENDS in vehicles and public areas, it is recommended that policymakers consider enacting stricter regulations on ENDS use in public spaces, similar to smoke-free laws for regular tobacco products, to protect public health from the risks of secondhand exposure.

## Figures and Tables

**Table 1:** Descriptive statistics of silicone wristband nicotine and urinary cotinine stratified by vaping conditions (N = 12, 6 ENDS users and 6 ENDS non-users).

	Overall ^b^			Month 1			Month 2			Month 3		
	ENDS User	Non-ENDS User	*p*-Value	ENDS User	Non-ENDS User	*p*-Value	ENDS User	Non-ENDS User	*p*-Value	ENDS User	Non-ENDS User	*p*-Value
**Silicone Wristband** **Nicotine (ng/g) ^a^**			0.0005			0.0303			0.0411			0.2468
Geometric Mean	324.1	27.9		488.9	37.1		415.7	37.8		159.3	15.6	
95% Confidence Limit	195.3–537.8	12.9–60.8		281.4–849.4	8.7–158.2		229.8–752.1	6.6–216		46.3–548.5	7.1–34.2	
Minimum	28.6	4.4		231.21	5.79		158.25	4.8		28.6	4.44	
Median	423.2	17.2		607.4	28.2		423.2	78.7		120.3	13.3	
Interquartile Range (IQR)	199.2–669.1	6.5–128.0		290.8–624.2	9.3–213.6		260.6–682.1	5.4–175.5		28.5–665.7	8.7–32.7	
Maximum	1125.6	641.6		1095.7	376.3		1125.6	641.6		679.4	61.2	
Geometric Standard Deviation	2.8	5.4		1.9	6.1		2.1	8.8		4.1	2.7	
**Urinary Cotinine (ng/mL) ^a^**			<0.0001			0.005			0.005			0.0049
Geometric Mean	759.3	1.7		846	1.4		753	2.9		686	1.2	
95% Confidence Limit	474.0–1215.0	1.1–2.7		327.9–2188.4	0.9–2.1		243.6–2315.0	0.6–13.7		234.0–2017.0	1.0–1.4	
Minimum	118	1		169.6	1		166.8	1		118	1	
Median	1013.0	1.3		1133.9	1.3		959	1.4		921.2	1.2	
Interquartile Range (IQR)	442.0–1490.0	1–1.4		658.6–1490.5	1.1–1.4		370.1–1937.0	1.1–6.8		471.0–1395.0	1.0–1.4	
Maximum	2205.2	36.1		2024.4	2.8		2205.2	36.1		1901.4	1.4	
Geometric Standard Deviation	2.6	2.5		2.5	1.5		2.9	4.3		2.8	1.2	

^a^ Represents using the Wilcoxon test, ^b^ includes all data regardless of the monthly time point.

## Data Availability

The datasets generated and/or analyzed during the current study are not publicly available due to the small sample size, which can make participants easily identifiable and thus raises concerns regarding confidentiality.

## Abbreviations

The following abbreviations are used in this manuscript:

ENDS	Electronic nicotine device systems

## Appendix A

**Table A1:** Individual self-reported physical areas of exposure by month.

	Past Seven Days of Exposure for Month 1, 2, 3																		Past 24 Hours of Exposure for Month 1, 2, 3																	
	Where You Live			Friend’s Home			Relative’s Home			In a Vehicle			Public Area			Somewhere Else			Where You Live			Friend’s Home			Relative’s Home			In a Vehicle			Public Area			Somewhere Else		
Month	1	2	3	1	2	3	1	2	3	1	2	3	1	2	3	1	2	3	1	2	3	1	2	3	1	2	3	1	2	3	1	2	3	1	2	3
**Non-ENDS users**																																				
1	Y	Y	Y		Y					Y	Y	Y		Y	Y		Y		Y	Y	Y							Y	Y				Y			
2		Y		Y	Y	Y	Y	Y		Y	Y	Y	Y	Y	Y	Y						Y	Y	Y		Y		Y	Y	Y	Y	Y	Y	Y		
3	Y	Y	Y	Y	Y	Y	Y	Y	Y	Y	Y	Y	Y	Y	Y	Y	Y	Y		Y	Y		Y	Y			Y			Y					Y	
4	Y	Y	Y	Y		Y			Y	Y	Y	Y	Y	Y	Y		Y	Y	Y	Y	Y			Y				Y	Y	Y					Y	
5	Y	Y	Y	Y	Y	Y				Y	Y	Y	Y	Y	Y	Y			Y	Y	Y							Y	Y	Y	Y					
6				Y		Y				Y	Y	Y	Y	Y	Y		Y	Y										Y	Y		Y					Y
Total response (%) ^a^	13 (72.2)			14 (77.8)			6 (33.3)			18 (100.0)			17 (94.4)			10 (55.6)			11 (61.1)			6 (33.3)			2 (11.1)			14 (77.8)			6 (33.3)			4 (22.2)		
**ENDS Users**																																				
1		Y	Y								Y			Y			Y																			
2	Y	Y	Y	Y	Y	Y				Y	Y	Y	Y	Y	Y	Y	Y	Y		Y	Y		Y	Y					Y	Y		Y	Y			
3		Y		Y	Y	Y	Y	Y	Y	Y	Y	Y	Y							Y								Y	Y			Y				
4				Y		Y				Y		Y	Y	Y	Y	Y								Y						Y		Y				
5	Y	Y	Y	Y	Y	Y				Y	Y	Y	Y	Y	Y			Y	Y	Y		Y	Y	Y				Y		Y	Y		Y			Y
6	Y	Y	Y	Y	Y	Y			Y	Y	Y	Y	Y	Y	Y	Y	Y		Y	Y	Y	Y	Y	Y		Y	Y	Y	Y	Y	Y	Y	Y			
Total responses (%) ^a^	12 (66.7)			14 (77.8)			4 (22.2)			15 (83.3)			14 (77.8)			8 (44.4)			8 (44.4)			9 (50.0)			2 (11.1)			10 (55.6)			9 (50.5)			1 (5.6)		

^a^ Total number of “yes” responses and corresponding percentages across all time points for each exposure location.
